# Computational Design of a Novel VLP-Based Vaccine for Hepatitis B Virus

**DOI:** 10.3389/fimmu.2020.02074

**Published:** 2020-08-27

**Authors:** Saeed Mobini, Milad Chizari, Ladan Mafakher, Elmira Rismani, Elham Rismani

**Affiliations:** ^1^Department of Immunology, School of Public Health, Tehran University of Medical Sciences, Tehran, Iran; ^2^Department of Medical Biotechnology, School of Allied Medical Sciences, Iran University of Medical Sciences, Tehran, Iran; ^3^Medicinal Plant Research Center, Ahvaz Jundishapur of Medical Science, Ahvaz, Iran; ^4^Department of Biology, Payam Noor University, Tehran, Iran; ^5^Molecular Medicine Department, Pasteur Institute of Iran, Tehran, Iran

**Keywords:** hepatitis B virus, epitope, vaccine, virus-like particles, molecular dynamics

## Abstract

Hepatitis B virus (HBV) is a global virus responsible for a universal disease burden for millions of people. Various vaccination strategies have been developed using viral vector, nucleic acid, protein, peptide, and virus-like particles (VLPs) to stimulate favorable immune responses against HBV. Given the pivotal role of specific immune responses of hepatitis B surface antigen (HBsAg) and hepatitis B core antigen (HBcAg) in infection control, we designed a VLP-based vaccine by placing the antibody-binding fragments of HBsAg in the major immunodominant region (MIR) epitope of HBcAg to stimulate multilateral immunity. A computational approach was employed to predict and evaluate the conservation, antigenicity, allergenicity, and immunogenicity of the construct. Modeling and molecular dynamics (MD) demonstrated the folding stability of HBcAg as a carrier in inserting Myrcludex and “a” determinant of HBsAg. Regions 1–50 and 118–150 of HBsAg were considered to have the highest stability to be involved in the designed vaccine. Molecular docking revealed appropriate interactions between the B cell epitope of the designed vaccine and the antibodies. Totally, the final construct was promising for inducing humoral and cellular responses against HBV.

## Introduction

There is approximately 257 million people worldwide suffering from hepatitis B infection. Hepatitis B virus (HBV) causes 887,000 deaths per year due to cirrhosis and hepatocellular carcinoma (HCC) (1). In spite of the current success in vaccines, hepatitis B disease challenges persist for various reasons. Firstly, 5–10% of the population remain without vaccine response (non-responders, <10 IU/L). Further, the dose of the antibody produced in most vaccinated individuals is not fully protective (low responders, <100 IU/L) (2). Another reason is the emergence and spread of vaccine-induced immune escape mutants that are resistant to the effects of the existing vaccines (3). Finally, there is lack of effective treatment for chronic hepatitis B patients (4). Accordingly, the development of a new vaccine will be required to enhance seroprotection in non-responders to current vaccines and to provide therapeutic immunization for patients with chronic HBV infection.

Hepatitis B virus is an enveloped virion with an icosahedral nucleocapsid core comprising a partially double-stranded circular DNA genome. The genome includes four genes that encode five proteins (5). The envelope proteins [hepatitis B surface antigen (HBsAg), 388–400 amino acids (aa)] encoded by the S gene contains three regions of pre-S1 (107–119 aa), pre-S2 (55 aa), and S (226 aa). Myrcludex (residues 2–48) is a highly conservative part of the pre-S1 region that triggers specific binding to the HBV receptor, sodium taurocholate co-transporting polypeptide (NTCP), in hepatocytes. Further, it induces antibodies that neutralize immune escape mutants. In other words, any mutation in this region leads to a missing virus binding ability to its receptor (6). Myrcludex can also bypass non- (or low) response to the S region (7). Likewise, the “a” determinant located at 124–147 amino acids in the S region of HBsAg is common in all serotypes and genotypes in HBV (8). Although the “a” determinant is capable of producing neutralizing antibodies against HBV, the spread of mutations in P120E, T123N, T126N, Q129L, Q129H, M133L, K141E, P142S, D144A, G145R, and N146S has diminished its efficiency (9). The receptor binding potential and the antibody-mediated neutralization effect of Myrcludex, as well as the high capability of immunodominant epitopes of the “a” determinant, have spiked some interest to generate new HBV vaccines.

Hepatitis B core antigen (HBcAg) is a 183- to 185-residue polypeptide chain from the C gene. Structurally, the HBcAg monomer is designated as an antiparallel helical protein connected by a loop that forms the spike feature of capsid. The spike tip of the virus, the major immunodominant region (MIR; amino acids 76–82), is known as the insertion site of foreign epitopes to develop the recombinant HBcAg particles owing to its role in inducing antigen-specific antibodies (10). The icosahedral capsid of HBV is composed of the self-assembly HBcAg dimer proteins that form T = 3 or T = 4 symmetry (11). Interestingly, in a lack of any homology to human proteins, HBcAg is introduced as an ideal therapeutic target (12). Further, HBcAg, as a virus-like particle (VLP), can bypass non- (or low) response to HBsAg in the current vaccines (13) while also containing CD4^+^ and CD8^+^ T cell epitopes (14). CD8^+^ T cells have HLA-A2 supertype epitopes in HBcAg that cover most allele HLA-A (HLA-A^∗^0201) in the world’s population (15). Recently, VLP-based vaccines which include HBcAg as a carrier are remarkable candidates for the insertion of heterologous fragments in the MIR region (14).

Several therapeutic HBV vaccines hold promise to target multi-epitopes involved in cellular and humoral immunity. A promising vaccine candidate with convenient immunological properties would be achieved by integrating proper fragments of HBsAg and HBcAg, given their crucial role in priming neutralizing antibodies and T cells activation, respectively. To this aim, a construct that contains B cell epitopes (including the “a” determinant and Myrcludex) for the generation of neutralizing antibodies and T cell epitopes (HBcAg) for the stimulation of helper and cytotoxic T cell responses can overcome the challenges. We implemented a computational approach to design an HBcAg-based VLP vaccine containing more immunogenic domains of HBsAg to stimulate broad and specific B and T cell responses against HBV. Briefly, the full three-dimensional (3D) structures of HBsAg and HBcAg were predicted and evaluated due to the lack of an experimentally determined conformation. Then, the construct containing the HBcAg dimer as the backbone and inserting fragments of HBsAg in MIRs was generated and assessed to retain the spike feature of VLP. Finally, the antigenicity, allergenicity, and immunogenicity of the ultimate construct were determined *in silico*.

## Materials and Methods

### Multiple Sequence Alignment and Phylogenic Studies

To find conserved residues and regions in the large envelope protein of HBV (HBsAg), multiple sequence alignment (MSA) was carried out using Clustal Omega [Research Resource Identifier (RRID): RRID: SCR_001591] at default parameters. Clustal Omega performs MSA using seeded guide trees and hidden Markov model (HMM) profile–profile techniques to generate alignments (16). The reviewed sequences retrieved from Uniprot were subjected to MSA. The lengths of these sequences ranged from 328 to 400 aa. Since the D genotype is the most prominent genotype in Iran and in the Middle East countries (17, 18), MSA was also implemented on the D genotype sequences (389 aa). The results of both alignments were visualized by Jalview (RRID: SCR_006459) (19). Moreover, a phylogenetic tree was built using MEGA7 (RRID: SCR_000667) (20).

### Secondary and Tertiary Structure Prediction and Validation

The secondary structures of the large envelope protein (HBsAg; isoform L, genotype D, UniProt ID: P03138) and core antigen (HBcAg; UniProt ID: Q67855) were predicted using PHD and NetSurfP web servers. PHD (RRID: SCR_018778) is a neural network system which uses evolutionary information of a protein to predict an accurate enough 2D structure as the initial point of 3D modeling (21). The sequence-based approach of NetSurfP-2.0 (RRID: SCR_018781) could promote accuracy in predicting the geometrical features of proteins by integrated deep learning (22).

For tertiary structure prediction, structural alignment for the primary sequences of HBsAg and HBcAg was performed using InterPro Scan and HHpred at their default parameters to find homologous structures. HHpred (RRID: SCR_010276) provided the query-template alignment using the PDB_mmCIF70 database (23). The results introduced the crystal structure of HBcAg as reported by 24. Although the sequence identity of the template (PDB ID: 1QGT) with HBcAg was defined to be 94%, the amino acid coverage of the 3D structure was related to the assembly domain of the protein in the N-terminal (residues 1–140). There was no template for the nanopeptides (residues 141–149) and the protamine domain in the carboxy terminal (residues 150–183). Thus, a combination of *ab initio* and homology modeling was used for modeling the entire HBcAg proteins.

Likewise, as the appropriate templates for HBsAg were only found for a few numbers of the residues in the N-terminal, the *ab initio* modeling was applied for protein structure prediction. Even if the online QUARK server (RRID: SCR_018777) allows a sequence length less than 200 amino acids, according to the community-wide critical assessment of protein structure prediction (CASP) experiments, its fragment-based assembly approach generates high-quality models for proteins with no homologous templates (25, 26). In this regard, the sequences of 1–200 of the N- and C-terminals of protein, including Myrcludex and the “a” determinant regions, were subjected to the QUARK server using default options to construct the 3D protein model, separately. Then, homology modeling was performed to obtain the entire 3D structure of the HBsAg protein. Finally, the stereochemical and geometrical quality of the modeled 3D structures was evaluated and validated using several web tools, including Verify3D, ProSA, and RAMPAGE (27–29).

### Vaccine Design Process

The sequences of various lengths of the C- and N-terminal pre-S1 and S domains of HBsAg (including Myrcludex and the “a” determinant) were inserted into the MIR (between residues 78 and 79) of each monomer of the dimer HBcAg protein. For conformational study of each VLPs-based vaccine, the 3D structure of the fusion particles was predicted through comparative homology modeling using the Modeller v9.19 program (RRID: SCR_008395) at default parameters (30). The predicted 3D structures of the HBsAg and HBcAg proteins were used as the template. For each fusion protein sequence, 10,000 generated models were ranked based on the discrete optimized potential energy (DOPE) score. Then, top 10 models were visualized and analyzed using PyMol software (RRID: SCR_000305) (31). The structural disorders in four helices of HBcAg due to the insertions were evaluated by aligning with the dimer HBcAg protein. A final model, as the designed VLP-based vaccine, was selected after stereochemical quality validation and optimization and was subjected to the following analysis.

### T Cell Epitope Prediction

The sequence of the designed VLP-based vaccine was conducted for T cell epitope prediction using the online prediction server Immune Epitope Database (IEDB; RRID: SCR_006604) (32, 33). Considering the associations of specific HLA allele variants with the outcome of HBV infection, three sets of HLA alleles were subjected to T cell epitope prediction. The first set was the most common MHC I (HLA-A^∗^0201)- and MHCII (HLA-DRB1)-restricted T cells in the general population. The second set was positive responders to HBV vaccination, including HLA-DRB1^∗^0101, HLA-DRB1^∗^0401, HLA-DRB1^∗^1301, and HLA-DRB1^∗^1501. The third set consisted of MHC I (HLA-B8)- and MHCII (HLA-DRB1^∗^0301, HLA-DRB1^∗^0701, and HLA-DQB1^∗^0201)-restricted T cells which were associated with non-responders to hepatitis B vaccine (34, 35). The default parameters of the prediction tools were set to predict the MHC-I and MHC-II epitopes as nanopeptides and 15-mer peptides, respectively.

### Prediction of B Cell Epitopes

Linear B cell epitopes of the designed VLP-based vaccine were predicted using the IEDB database. Certain features of the protein correlated with continuous antibody epitopes (hydrophilicity, flexibility, accessibility, turns, exposed surface, polarity, and antigenicity) were analyzed using Parker hydrophilicity prediction, Karplus and Schulz flexibility scale, Emini surface accessibility scale, Chou and Fasman beta turn prediction, and Bepipred methods, respectively (36–40). Further, the Ellipro method in the IEDB database was used to identify discontinuous B cell epitopes based on solvent accessibility and flexibility (41).

### Evaluating the Antigenicity and Allergenicity of the Designed VLP-Based Vaccine

The allergenicity of the designed VLP-based vaccine was predicted using the AlgPred (RRID: SCR_018780) and AllerTOP web servers (RRID: SCR_018496) (42, 43). Estimation in hybrid approach of AlgPred, which is a combination of support vector machine (SVM), IgE epitope, blast search on allergen representative peptides (ARPs BLAST), and Motif Alignment and Search Tool (MAST), allowed a reliable allergenic assay of proteins. Likewise, AllerTOP implemented the *k*-nearest neighbors (*k*-NN) as a machine learning method for the classification of allergens with 85.3% accuracy.

Further, the antigenicity of the designed VLP-based vaccine was evaluated using the VaxiJen v2.0 (RRID: SCR_018514) and ANTIGENpro (RRID: SCR_018779) web servers (44, 45). The free online VaxiJen 2.0 server classifies antigens considering the physicochemical properties of the protein in an alignment-independent manner. ANTIGENpro predicts the protein’s antigenicity using a sequence-based method and machine learning classifier as a trained predictor *via* protein microarray analysis. The default parameters of the prediction tools were set to estimate the antigenicity and allergenicity.

### Immune Simulation

Computational immune simulation was performed using the C-ImmSim server (RRID: SCR_018775^1^) to characterize the immune response profile of the designed VLP-based vaccine (46). This server implements machine learning techniques for the prediction of immune interactions through simultaneously simulating three anatomical regions found in mammals: the bone marrow, the thymus, and the lymph node. In accordance with the routine administration schedule for the hepatitis B vaccine in adults, three injections were given at 0, 1, and 6 months (47). The simulation parameters were set at default with time steps set at 1, 90, and 540 (each time step is 8 hours). The results of the cellular and humoral response were interpreted from the plots.

### Molecular Dynamics Simulations

To investigate the conformational stability of the designed VLP-based vaccine under physiological conditions, molecular dynamics (MD) simulations were performed and compared to the HBcAg. MD simulations were implemented in GROMACS 5.1.5 (RRID: SCR_014565) using Amber99SB-ILDN force field (48). The system was prepared with the SPC/E water model in a cubic solvation box with a distance of 1.0 nm and then neutralized with Na and Cl ions. The structure was relaxed by energy minimization using the steepest descent algorithm with a maximum force constraint of 1,000 kJ mol^–1^ nm^–2^. The long-range electrostatic interactions were calculated with the particle mesh Ewald (PME) method. The Linear Constraint Solver (LINCS) algorithm was used to constrain bonds. Prior to the MD simulation, the temperature of the system was stabilized under an NVT ensemble (constant number of particles, volume, and temperature) at 310 K for 100 ps with the Berendsen thermostat method. Then, the system was equilibrated under constant number of particles, pressure, and temperature (NPT) with the pressure coupling of Parrinello–Rahman for 100 ps. MD simulations were run for a period of 100 ns with 2-fs time steps. The trajectories of the MD simulations were analyzed for root-mean-square deviation (RMSD), root-mean-square fluctuation (RMSF), and radius of gyration (RoG).

### Molecular Docking

Experimentally, the X-ray structure is a suitable way to obtain the conformation of the antibody–antigen complex as well as atomic interactions on their interface. Nowadays, computational docking provides a fast and inexpensive route to obtain reasonable results (49). The molecular interactions between the designed VLP-based vaccine and the anti-HBsAg antibodies were studied using the ClusPro web server (RRID: SCR_018248) in the antibody mode (50). This web server predicts the antibody–antigen complexes using a combination of Decoys as the Reference State (DARS) and the docking program PIPER based on the fast Fourier transform (FFT) correlation approach. The 3D structures of the anti-HBsAg antibodies were obtained from PDB (PDB IDs: 1H3P, 4Q0X, and 5YAX). The complementarity determining regions (CDRs) of the antibodies were defined by the Kabat, Chothia, and IMGT numbering schemes using the Structural Antibody database (SAbDab) (51). The complexes were visualized using PyMOL software and analyzed using Ligplot+ software (RRID: SCR_018249) (52).

## Results

### HBsAg, Combination of Conserved Sequence, and Various Evolutionary Genotypes

**FIGURE 1:**
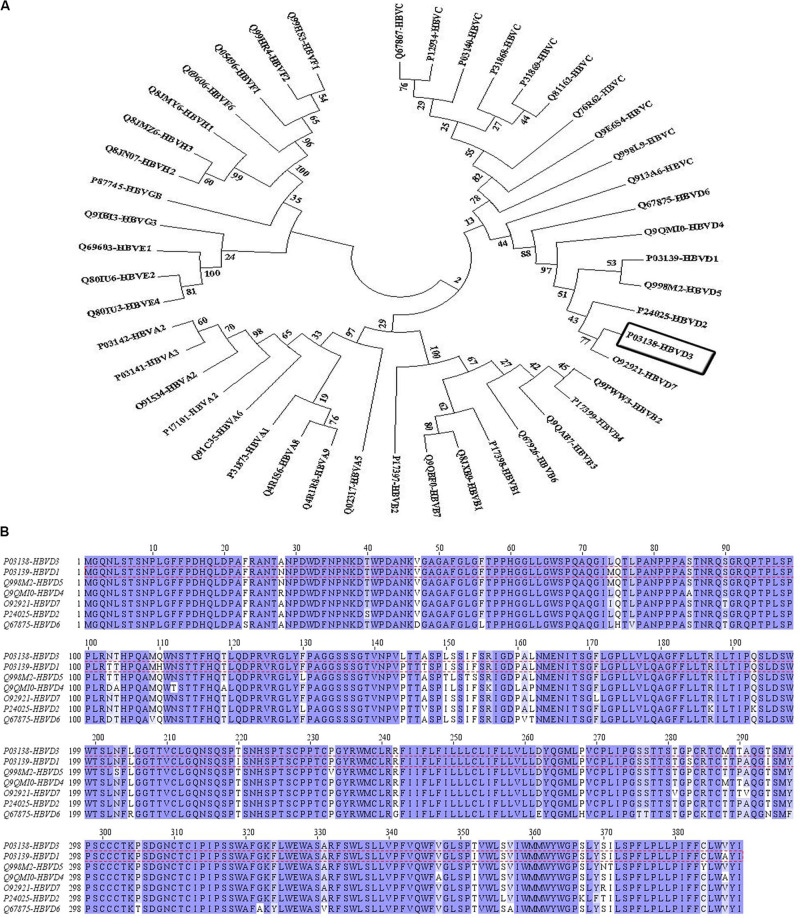
The evolutionary relation was derived using the neighbor-joining method and multiple sequence alignment of hepatitis B surface antigen (HBsAg) among hepatitis B virus (HBV) genotype D. **(A)** The bootstrap consensus tree concluded from 1,000 replicates was taken to represent the evolutionary relation of the analyzed sequence of the large envelope protein of various genotypes of HBV. The percentage of replicate trees in which the associated proteins clustered together was visible *above the branches*. The evolutionary distances were computed using the Poisson correction method. The analysis involved 47 amino acid sequences. Evolutionary analyses were conducted in MEGA7. **(B)** The sequences have been colored by conservation based on the Blosum62 matrix in Jalview. The most dominant genotype of HBsAg in Iran, genotype D1, is depicted by *red dashed lines*.

Multiple sequence alignment was carried out to find the conserved regions among the large envelope protein of various genotypes of HBV (HBsAg). Although the MSA results revealed higher conservation in the whole sequence of HBsAg among the genotypes (Supplementary Figure S1), it is reported that the pre-S1 and S domains of HBsAg represented the adaptive evolutionary feature which provides heterogeneous viral genotypes. This hallmark could be interpreted given the pivotal role of these domains of HBsAg in viral replication and infection in the host (53). The results have been presented in a phylogenetic tree with the neighbor-joining (NJ) method in MEGA7 (Figure 1A). The bootstrap consensus tree concluded from 1,000 replicates was taken to represent the evolutionary relation of the analyzed sequences of the large envelope protein in various genotypes of HBV. The percentage of replicate trees in which the associated proteins clustered together was assigned above the branches. The evolutionary distances were computed using the Poisson correction method. The analysis involved 46 amino acid sequences. The phylogenetic tree showed a close relationship between HBsAg of genotypes A and B, and then C and D, while in genotypes E, F, and G, it was more divergent from the other genotypes. Furthermore, MSA of the HBsAg sequences among the D genotype, which is illustrated by conservation based on Blosum62 matrix, showed high conservation in most regions. However, a few amino acid differences in their sequence led to sub-genotypes D1–D7 (Figure 1B).

### Prediction and Validation of HBsAg and HBcAg Structures

**FIGURE 2:**
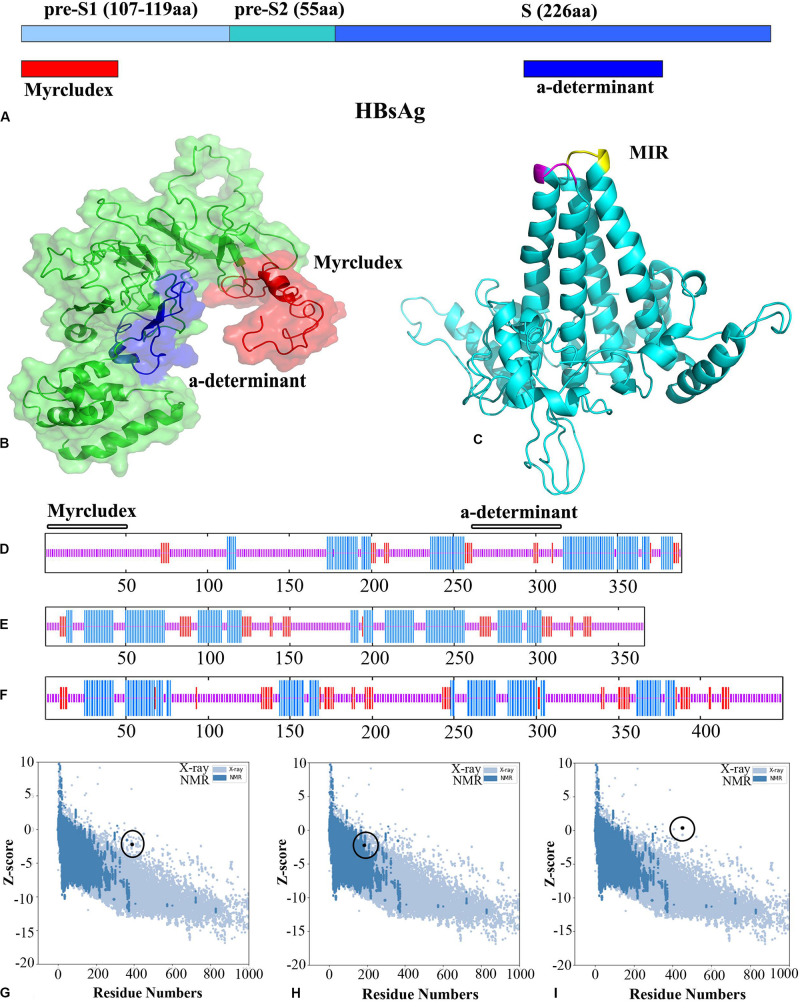
Hepatitis B surface antigen (HBsAg) and hepatitis B core antigen (HBcAg) representations, the *Z*-score ProSA plots of the structures, and their cartoonic representation. **(A)** Schematic view of the S gene in HBV. **(B)** Three-dimensional (3D) structure of an HBsAg (residues 1–389). Myrcludex and the “a” determinant regions are shown in *red* and *blue*, respectively. **(C)** 3D structure of the HBcAg dimer (the MIR in monomers is shown in *magenta* and *yellow*). **(D–F)** Secondary structure prediction of HBsAg, HBcAg dimer, and the designed virus-like particle (VLP)-based vaccine. The alpha helix, extended strand, and random coils are indicated in *blue*, *red*, and *pink lines*, respectively. **(G–I)** ProSA plots of HBsAg, HBcAg, and the VLP-based vaccine. *Black spot* represents the evaluation of the input structure within the range of experimentally determined structure for proteins of similar size.

In parallel with MSA, the secondary structures of HBsAg and HBcAg were analyzed to determine the structural content of the amino acid sequence as alpha helix, extended strand, turn, and coil. The result is depicted in Figure 2 and summarized in Table 1. The conformational content predicted for the HBcAg protein was consistent with its crystallographic structure, and as expected, much of its structure was composed of the alpha helix. In contrast, the coiled and alpha helix contents in the HBsAg protein were tantamount, which should be noted when predicting the third structure. Likewise, the structural feature of the HBsAg protein, including the surface accessibility as well as residue disorders and phi–psi dihedral angles of the amino acids in the sequence, was investigated and depicted in Supplementary Figure S2.

**TABLE 1:** Secondary and tertiary structure validations.

	ProSA *Z*-score	RAMPAGE			Verify3D score (%)	PHD		
				
		Favored (%)	Allowed (%)	Outlier (%)		Alpha helix	Extended strand	Random coil
HBcAg	−2.23	89.2	8.6	2.2	82.23	51.08	11.35	37.57
HBsAg	−4.76	80.7	15.7	3.6	80.05	39.46	10.9	49.64
VLP-based vaccine	−2.26	85.6	11.2	3.2	80.17	45.63	12.78	41.59

After evaluating the predicted model by QUARK for the nanopeptides (residues 141–149) and the protamine domain in the carboxy terminal (residues 150–183) of HBcAg, homology modeling was performed using Modeller v9.19 software. A total of 10,000 generated models were ranked based on the DOPE score to find the top 10 models for conformational analysis. Likewise, the 3D model related to the N- and C-terminal domains of HBsAg was obtained from QUARK. The predicted models were introduced to Modeller as templates in order to design the entire HBsAg. The overall quality of the final models of proteins was rated by *Z*-score in ProSA, which illustrated the quality of the predicted models compared to the experimentally validated structure of the proteins (X-ray and NMR) (Figure 2). The torsional angles of the protein backbone were analyzed in the predicted models by the Ramachandran plot. Although there were 2.2 and 3.6% outlier residues in the predicted models of HBcAg and HBsAg, respectively, the high amounts of residues located in the favored and allowed regions confirmed the quality of the modeling. As the results of the secondary structure predictions were compatible with the 3D models, Verify3D scores also designated the adaptability of the 3D–1D structures. A score higher than 80% is denoted as the remarkable amount of amino acids with a score ≥0.2 in the 3D/1D profile (Table 1).

### Structural Analysis of the VLP-Based Vaccine

**FIGURE 3:**
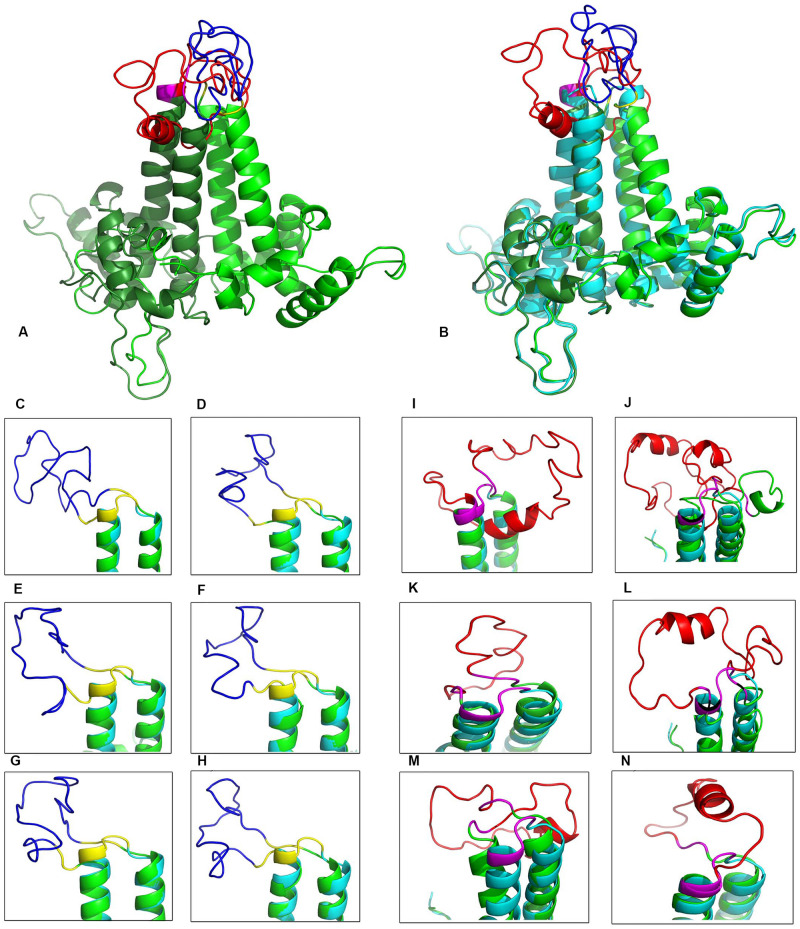
Structural view of the designed virus-like particle (VLP)-based vaccine. **(A)** Overview of final stable VLP-based vaccine [insertion of 1–50 of the Myrcludex region of HBsAg in the first monomer of HBcAg (*red*) and 118–150 of the “a” determinant of HBsAg in the second monomer (*blue*)]. **(B)** Structural alignment of the VLP-based vaccine and the HBcAg dimer in *green* and *cyan*, respectively. **(C–H)** Close-up view of various “a” determinant insertions in the immunodominant region (MIR) of the second monomer (118–150, 123–150, 121–147, 124–147, 121–148, and 123–148, respectively). **(I–N)** Close-up view of a range of Myrcludex insertions in the MIR of first the monomer (1–50, 2–48, 1–21, 9–48, 18–48, and 20–48, respectively).

In order to preserve the self-assembly feature of the α-helical HBcAg, conformational disorders due to the insertion of heterolength epitopes in the MIR loops were evaluated precisely. As shown in Figure 3, generating models by inserting various lengths of the Myrcludex region (1–50) of HBsAg resulted in divergent perturbations of the α-helical structure of the HBcAg protein. The minimum disarrangement was related to the insertion of the entire sequence of Myrcludex. Similarly, the effect of different fragments of the “a” determinant region of HBsAg was determined on the stability of the helical capsomere. Note that the change of even a single amino acid in the fragment length of the “a” determinant disrupted the HBcAg capsid helix structure. The insertion of an extended length of the “a” determinant has led to the minimum irregularity of the carrier capsid structure. Finally, specific protein fusion was constructed by inserting regions 1–50 of Myrcludex in the first monomer and regions 118–150 of HBsAg in the second monomer of dimer HBcAg. The stereochemistry and geometry of the final VLP were investigated following MD simulation analysis of model (Table 1).

### T Cell Epitope Identification on the VLP-Based Vaccine

The final sequence of the designed VLP-based vaccine was analyzed to look for the sequence of T cell epitopes. The potent MHC I-restricted cytotoxic T cell epitopes were predicted using the IEDB recommended 2.18 [artificial neural network (ANN), stabilized matrix method (SMM), and combinatorial peptide libraries (CombLib)], NetMHCpan4.0, NetCTLpan, and MHC-NP servers. The 15-mer MHC II binding epitopes were collected using the IEDB recommended 2.22 (NN-align, SMM-align, CombLib, and Sturniolo) and NetMHCIIpan3.2 servers. The predicted output of each allele set was sorted by percentile rank to find sequences with higher affinity of binding. The range of percentile rank ≤1 (IC_50_ values <50 nM) was reported as high-affinity peptide sequences, while the percentile rank ≤10 (IC_50_ values <500 nM) was considered as intermediate affinity of binding. T cell-predicted epitopes on the full sequence form of fusion candidates are listed in Table 2.

**TABLE 2:** Predicted MHC class I and II epitopes of the designed virus-like particle (VLP)-based vaccine.

HLA sets in HBV	MHC	Allele	Position	Sequence	Percentile rank*
Common	MHC I	HLA-A*02:01	18–27, 253–262	FLPSDFFPSV	0.11
	MHC II	HLA-DRB1*01:01	163–177, 382–396	ETVLEYLVSFGVWIR	4.4
Non-responder	MHC I	HLA-B*08:01	214–222, 222–230, 431–444, 441–449	SPRRRRSQS	0.3
			207–215, 426–434	SPRRRTPSP	0.2
			150–158, 369–377	LLWFHISCL	0.5
			142–150, 361–369	NMGLKIRQL	1
	MHC II	HLA-DRB1*03:01	20–34, 255–269	PSDFFPSVRDLLDTA	7.1
		HLA-DRB1*07:01	162–176, 381–395	RETVLEYLVSFGVWI	2.8
			144–158, 363–377	GLKIRQLLWFHISCL	7
		HLA-DQB1*02:01	11–25, 246–260	ATVELLSFLPSDFFP	0.77
			5–19, 240–254	PYKEFGATVELLSFL	0.92
			13–27, 248–262	VELLSFLPSDFFPSV	0.94
			10–24, 245–259	GATVELLSFLPSDFF	0.97
Positive responsive	MHC II	HLA-DRB1*13:01	163–177, 382–396	ETVLEYLVSFGVWIR	0.53
			169–183, 388–402	LVSFGVWIRTPPAYR	0.53
		HLA-DRB1*15:01	163–177, 382–396	ETVLEYLVSFGVWIR	0.84
		HLA-DRB1*04:01	132–146, 351–365	RDLVVNYVNTNMGLK	2.5

*Percentile rank ≤1: high-affinity peptide sequences with the antigenic property; percentile rank ≤10: intermediate-affinity peptide sequences with the antigenic property.

### Prediction of B Cell Epitopes Revealed the Potency of the Inserted Fragments of HBsAg

**FIGURE 4:**
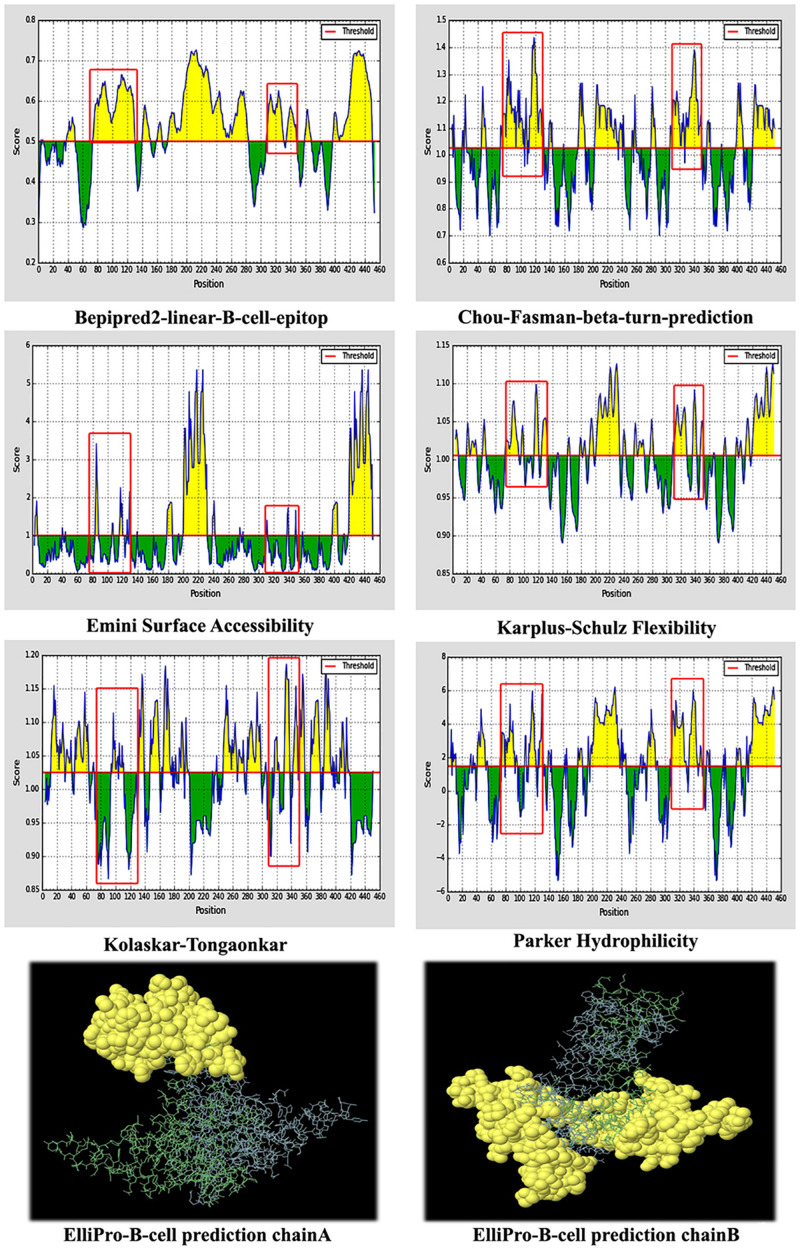
Predicted B cell epitopes of the designed virus-like particle (VLP)-based vaccine. **(A)** Linear B cell epitopes based on the various physicochemical properties of the residues. The *red boxes* are related to the first and second fragments of hepatitis B surface antigen (HBsAg) inserted into hepatitis B core antigen (HBcAg). **(B)** Predicted discontinuous B cell epitopes for each monomer of the designed vaccine.

The continuous antibody epitopes on the full length of the designed VLP-based vaccine were predicted using methods in the IEDB server. Considering the various physicochemical properties of the residues in the different mentioned methods, several linear sequences were delineated as B cell epitopes based on the scores above the default thresholds (Figure 4). Details on the location of the assigned epitopes indicated that the inserted fragments of HBsAg into HBcAg tend to be potent antibody epitopes. Further, discontinuous antibody epitopes were estimated using ElliPro based on the 3D structure of the designed vaccine. Firstly, Ellipro computed the shape of the protein as an ellipsoid. Then, the protrusion index (PI) of the residues was calculated as a measure of the atoms lying inside or outside the ellipsoid. Finally, the neighboring resides were clustered based on the PI scores. Higher scores were defined as more extensive solvent accessibility of the residues. The PI values of chains A and B were 0.724 and 0.672, respectively, which were above the minimum threshold of the program (default score, 0.5). The 3D view of the predicted discontinuous B cell epitopes for each monomer is depicted in Figure 4.

### The Antigenicity and Allergenicity of the Vaccine Candidate Were Predicted

The original sequence of HBcAg and the final sequence of the designed VLP-based vaccine were conducted to predict the allergenic property using the AllerTOP v.2 and AlgPred servers. Allergenicity was predicted based on the amino acid composition and mapping of the IgE epitope, which revealed that both sequences were non-allergens. Further, the antigenic trait of the designed vaccine (HBcAg including Myrcludex and the “a” determinant sequence of HBsAg) was estimated by VaxiJen 2.0 server to be 0.5449, with a virus model at thresholds of 0.4 and 0.498854 with ANTIGENpro. The scores for the original HBcAg were predicted to be 0.5245 and 0.359569 by the VaxiJen and ANTIGENpro servers, respectively. The results defined the sequences as probable antigens.

### Immune Simulation

**FIGURE 5:**
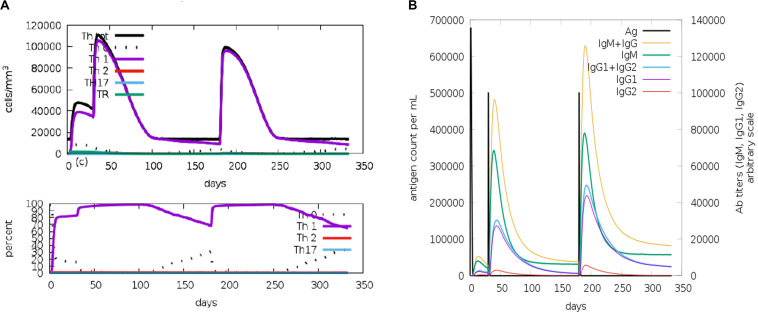
C-ImmSim representation of the immune stimulation of the designed virus-like particle (VLP)-based vaccine. **(A)** Phenotypic evaluation of T helper cells against a vaccine injection. **(B)** Generation of immunoglobulins in response to vaccine injections (0, 1, and 6 months). Specific subclasses are demonstrated as *colored peaks*.

The C-ImmSim model illustrates both the humoral and the cellular responses of a mammalian immune system to the presence of antigens at the cellular level. The results of the immune simulation indicated appropriate responses of cellular and humoral immunity to the designed VLP-based vaccine. The cellular immune response was determined by an increase of T helper 1 cells and no T regulatory response was detected (Figure 5A). The humoral response was characterized by high levels of IgM and IgG (IgG1 + IgG2) consequently of three vaccine injections (Figure 5B).

### The Designed VLP-Based Vaccine Was Hopefully Stable and Folded During MD Simulations

**FIGURE 6:**
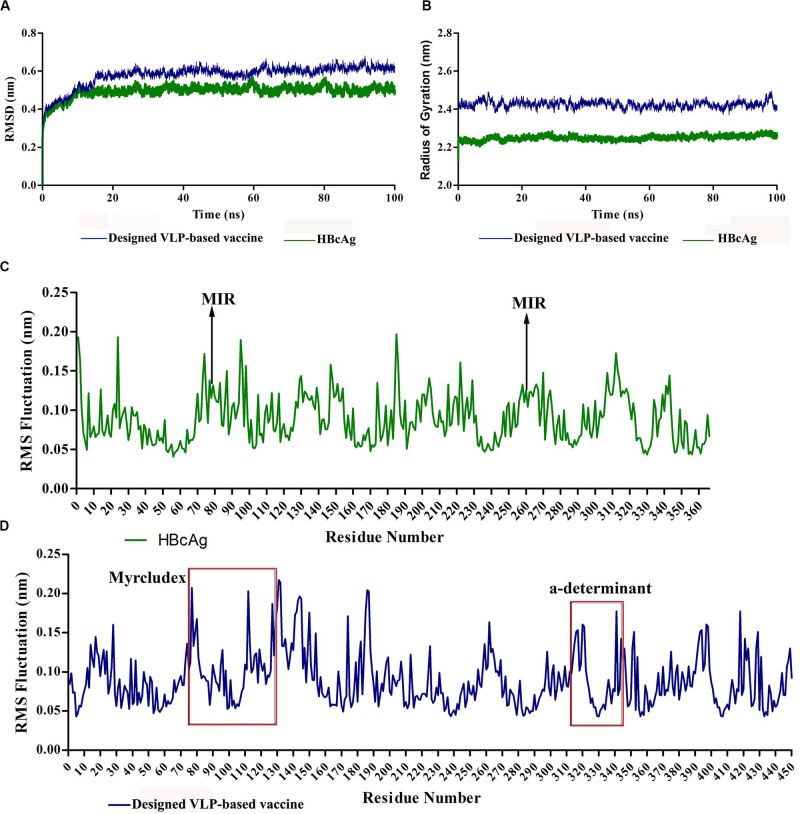
Molecular dynamics (MD) analysis of hepatitis B core antigen (HBcAg) and the designed virus-like particle (VLP)-based vaccine during 100-ns simulations. **(A)** Root-mean-square deviation (RMSD) plot of proteins indicating acceptable stability of the structures. **(B)** Radius of gyration (RoG) plot of proteins representing stable folding of the structures. **(C,D)** Root-mean-square fluctuation (RMSF) plots of HBcAg and the designed vaccine, respectively. The *red boxes* indicate the region of inserted fragments into HBcAg.

The conformational behavior of the designed vaccine during simulations was checked by calculating the backbone RMSD with respect to the initial structure. Figure 6A presents the results compared to the dimer-designed vaccine. Despite limited fluctuations during the simulations, the final structure of the protein has reached acceptable stability. The compactness and folding of the protein structure was investigated by the RoG (*R*_g_). The relatively stable diagram of this parameter during the simulation indicated the conservation of protein folding. This value for HBcAg and for the designed vaccine proteins showed similar ranges, which could be promising in preserving the 3D structure of the capsomeres after the addition of HBsAg fragments (Figure 6B). Analysis of the fluctuation of residues, *via* RMSF, indicated the residue’s movements during the simulation. The utmost of the amino acid displacements related to their initial positions were at the two ends of the proteins. However, some of the oscillations were associated with the regions where fragments of HBsAg were added to the capsomeres. The overall fluctuations in the two proteins were balanced and within the same range (Figures 6C,D).

### Molecular Docking

**FIGURE 7:**
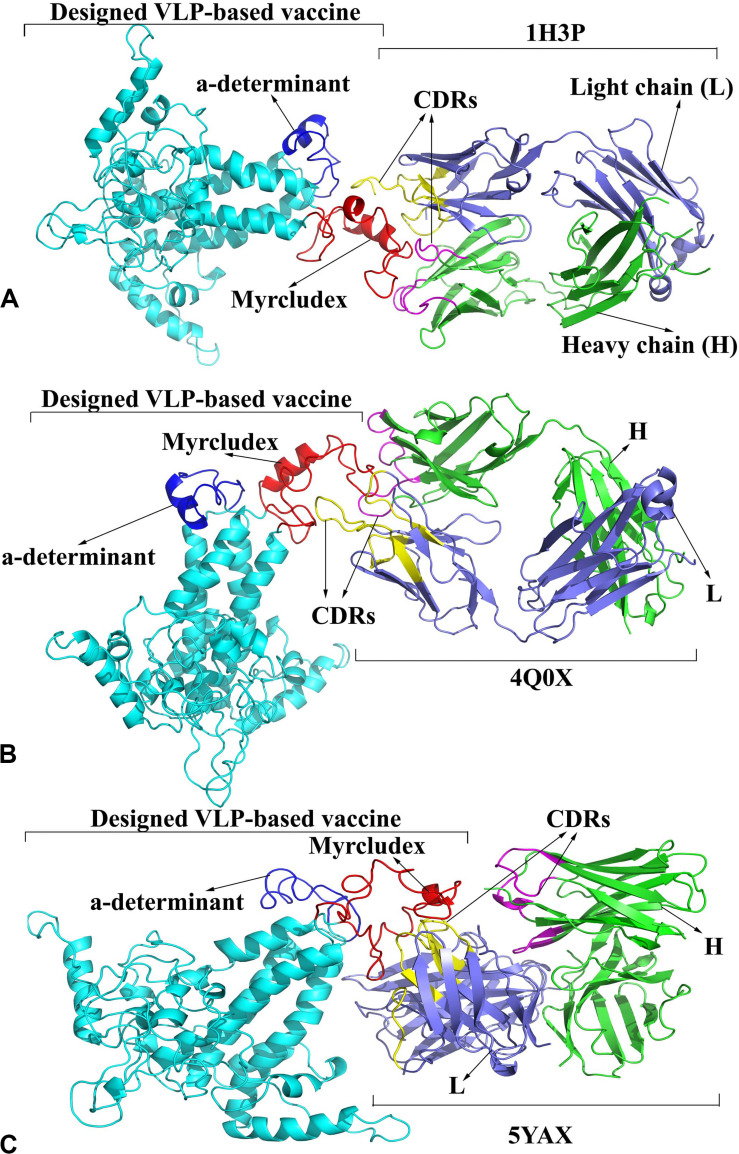
Binding model of the designed virus-like particle (VLP)-based vaccine and antibodies. **(A)** 1H3P-designed vaccine complex. **(B)** 4Q0X-designed vaccine complex. **(C)** 5YAX-designed vaccine complex. The heavy and light chains of antibodies are colored in *green* and *slate blue*, respectively. The complementarity determining regions (CDRs) of the H and L chains are displayed in *magenta* and *yellow*, respectively. The designed vaccine, Myrcludex, and the “a” determinant are reflected in *cyan*, *red*, and *dark blue*, respectively.

ClusPro docking server is recommended to provide a file consisting of non-CDRs of the antibody. The heavy and light chains of the 1H3P, 4Q0X, and 5YAX antibodies were analyzed in the SAbDab server using various numbering schemes (Kabat, Chothia, and IMGT) to determine CDR residues. Since these antibodies were anti-Myrcludex region of HBsAg, the molecular interactions of HBc VLPs were investigated focusing on the first monomer. However, there was no released 3D structure of the anti-“a” determinant region of the HBsAg antibody. The analysis revealed that the major residues of CDRs of the H and L chains were located in the loops of antibodies and cooperatively interacted with Myrcludex residues of the VLP-based vaccine. Details of these interactions in terms of the interacted residues and hydrogen bonds have been drawn up by Ligplot+ (Supplementary Figures S3–S5) and visualized by PyMol (Figure 7).

The bioinformatics tools that were used in the study and their descriptions are summarized in Table 3. The RRIDs were extracted from the Resource Identification Portal.

**TABLE 3:** Bioinformatics tools in the study.

Tools	Description	RRID
Clustal omega	Multiple sequence alignment (MSA)	SCR_001591
MEGA7	Phylogenetic tree	SCR_000667
PHD web server	Secondary structure prediction	SCR_018778
NetSurfP web server	Secondary structure prediction	SCR_018781
HHPred	Structural template alignment	SCR_010276
QUARK	*De novo* protein structure prediction	SCR_018777
Modeller	Comparative homology modeling	SCR_008395
PyMol	Molecular visualization software	SCR_000305
Immune Epitope Database (IEDB)	T cell, B cell epitope prediction	SCR_006604
AlgPred web server	Allergenicity prediction	SCR_018780
AllerTOP web server	Allergenicity prediction	SCR_018496
VaxiJen	Antigenicity prediction	SCR_018514)
ANTIGENpro	Antigenicity prediction	SCR_018779
C-ImmSim server	Immune simulation	SCR_018775
GROMACS	Molecular dynamics simulations	SCR_014565
ClusPro web server	Molecular docking	SCR_018248
Ligplot+ software	Generation of 2D protein–protein interaction diagrams from 3D coordinates	SCR_018249

## Discussion

In recent years, several therapeutic vaccination approaches have been developed for HBV to boost the immune system and ideally eradicate the virus. VLP-based vaccines include HBcAg as a carrier, a known candidate for the insertion of heterologous fragments in its MIR region (14). The aim of this study was to design an HBcAg-based VLP vaccine containing more immunogenic domains of HBsAg to induce multivalent immune responses.

We initially investigated the sequence conservation of HBsAg within known HBV types despite phylogenetic variations that led us to select two regions with an overall conserved sequence in the C- and N-terminals of protein. There are many plausible merits to explain the choice of these regions for incorporation into the final structure as a promising designed vaccine.

The main advantage of selecting these specific regions (“a” determinant and Myrcludex) of HBsAg rather than its entire structure in the vaccine construct is based on the roles of HBsAg in immunosuppression mechanisms in hepatitis B disease. HBsAg can inhibit innate immunity by blocking Toll-like receptor signaling pathways in Kupffer cells (KCs) and liver sinusoidal endothelial cells (LSECs) (54, 55). Also, HBsAg can defect dendritic cells, which are known as the most important antigen-presenting cells (APCs) (56). HBsAg may impair CD8^+^ T cell responses through persistent antigen stimulation (57–59). It can repress T cell responses by stimulating the differentiation of monocytes into myeloid-derived suppressor cells (MDSCs) and boost the regulatory T cell response (60, 61). In this regard, the cellular immune profile of the designed VLP-based vaccine comprising the specific regions of HBsAg indicated an increase of T helper 1 cells and no T regulatory response.

Additionally, studies have shown a link between multiple sclerosis and the existing HBV vaccine, with the mechanism relying on the amino acid similarity between HBsAg and the myelin oligodendrocyte glycoprotein (MOG) or other myelin antigens (62). Further, HBsAg-specific CD8^+^ T cells may be an essential trigger to induce HBV-associated HCC (63).

Another advantage of this construct over the current vaccines was the coverage of escaped mutants. This feature was derived from the Myrcludex fragment where any mutation in this region reduces the pathogenesis of the virus (64). Also, antibodies produced against this construct can eliminate virus-infected cells using the antibody-dependent cellular cytotoxicity (ADCC) mechanism. The presence of HBcAg in this construct compared to the current vaccine allows the production of CD8^+^ T cell responses and, subsequently, the eradication of reservoirs of chronic HBV (65).

Exhausted T cells that are present in the chronic type of hepatitis B disease have been characterized as follows: decreased cytokine production, increased expression of inhibitory receptors such as PD1 and CTLA-4, etc. (66). It has been shown that core-specific CD8^+^ T cells have a less defected functionality than other specific CD8^+^ T cells due to their mildly exhausted phenotype. Thus, this finding demonstrates that core-specific CD8^+^ T cells can represent promoted responsiveness to PD1 pathway blockade (67, 68).

The CD4^+^ T cell epitopes on our vaccine construct enabled the induction of cytokines and signals required to strongly stimulate the adaptive immune response. Indeed, molecular analysis of HLA class II exhibited that high-affinity ligands for the diversity of class II alleles were more common for HBcAg than for the envelope antigens, particularly HBsAg. Studies have found that HBcAg is about 100-fold more immunogenic than HBsAg at both the T helper cell and B cell levels (35). Finally, allergenicity analysis of the designed vaccine indicated that it was neither an allergen nor it may cause any hypersensitivity reactions.

The designed vaccine relied on the self-assembling feature of HBcAg, which preserved VLP formation. Considering the importance of proper folding in the antiparallel helices of HBcAg forming the spikes, a vast range of insertion sequences of HBsAg have been evaluated to allow the least structural movement of the carrier. Finally, fragments of 1–50 at the N-terminal (Myrcludex) and 118–150 at the C-terminal (“a” determinant) passed the evaluations to preserve the backbone folding of HBcAg.

Regarding estimation of the cellular and humoral immunity due to the designed vaccine, T cell prediction as well as B cell identification showed acceptable results of the efficacy of the construct. Some HLA alleles have a positive influence on the immune response to hepatitis B vaccine, such as DRB1^∗^1301, DRB1^∗^1501, and DRB1^∗^0401. The results indicated that the designed vaccine can be identified by these alleles. The positive responsiveness of HLA class II elicits antibody production by activating T helper cells and subsequently stimulating B cells. Further, the presence of weak antibody responses to the common hepatitis B vaccine is attributed to HLA non-responders, such as HLA-B^∗^0801, DRB1^∗^0301, DRB1^∗^0701, and DQB1^∗^0201. The designed vaccine contained epitopes for these alleles and could bypass their effects. Consistent with the B cell stimulating potential of the designed VLP-based vaccine, the humoral response in the results of the immune simulation was specified by the high levels of IgM and IgG (IgG1 + IgG2) consequently of three vaccine injections.

In addition, the presence of epitopes identified by HLA class I enables the construct to eliminate hepatitis B-infected cells through the induction of cytotoxic T cells (CD8^+^ T cells) and related mechanisms such as IFN-γ, perforin, and granzyme, so it can cover chronic hepatitis B patients (therapeutic effect).

B cell epitope prediction revealed that the inserted HBsAg fragments represented their promising functionality to induce an anti-HB antibody response. These regions were detected as linear B cell epitopes in the construct. Also, to evaluate this feature, interactions of the designed vaccine with anti-HBsAg were studied by molecular docking. The analysis indicated that the designed vaccine specifically interacted with anti-Myrcludex in their CDRs, suggesting that B cell epitope related to HBsAg–Myrcludex was recognized by the antibodies. However, there was no released anti-“a” determinant 3D structure to assess the second insertion.

An ideal vaccine needs to be inducible for both neutralizing antibodies and cellular immunity. Along with the immunogenic role of HBsAg, HBcAg is reported to be well suited in priming B cells *via* the activation of T cells. A combination of HBsAg- and HBcAg-based vaccines has been evaluated in research of finding proper strategies for chronic hepatitis B patients. Their incorporation can form a highly immunogenic structure to enhance humoral immunity against both antigens (69). In our study, *in silico* assessments of the designed vaccine showed the feasibility of humoral and cellular immunogenicity while declaring no allergenicity effect. Taken together, the synergistic effect of HBsAg and HBcAg could be an appealing approach to developing a wider-spectrum vaccine against HBV in acute as well as in chronic cases.

## Conclusion

This study evaluated a computational strategy to develop a VLP-based vaccine against HBV by inserting HBsAg fragments into the MIR region of HBcAg. Although T cell/B cell epitope predictions as well as the immune response profile of the designed vaccine displayed defensible features in feasibly stimulating humoral and cellular immunity, we recommend *in vitro*/*in vivo* studies to determine therapeutic efficacy for the treatment of HBV infection.

## Data Availability Statement

The datasets presented in this study can be found in online repositories. The names of the repository/repositories and accession number(s) can be found in the article/ Supplementary Material.

## Author Contributions

SM, MC, and EHR contributed to the conception and designed the study. SM, MC, LM, EMR, and EHR collected and analyzed the data and assisted in the writing. EHR, SM, and MC approved the final manuscript. All authors contributed to the article and approved the submitted version.

## Conflict of Interest

The authors declare that the research was conducted in the absence of any commercial or financial relationships that could be construed as a potential conflict of interest.

## Footnotes

1 http://150.146.2.1/C-IMMSIM/index.php

## Abbreviations

ADCC: antibody-dependent cellular cytotoxicity
APCs: antigen-presenting cells
ARPs BLAST: Basic Local Alignment Search Tool on allergen representative peptides
CDRs: complementarity determining regions
DARS: Decoys as the Reference State
DOPE: discrete optimized potential energy
FFT: fast Fourier transform
HBcAg: hepatitis B core antigen
HBsAg: hepatitis B surface antigen
HBV: hepatitis B virus
HCC: hepatocellular carcinoma
HMM: hidden Markov model
IEDB: Immune Epitope Database
KCs: Kupffer cells
LINCS: Linear Constraint Solver algorithm
LSECs: liver sinusoidal endothelial cells
MAST: Motif Alignment and Search Tool
MD: molecular dynamics
MDSCs: myeloid-derived suppressor cells
MIR: major immunodominant region
MOG: myelin oligodendrocyte glycoprotein
MSA: multiple sequence alignment
NPT: constant number of particles pressure and temperature
NTCP: sodium taurocholate co-transporting polypeptide
NVT ensemble: constant number of particlesvolume and temperature
PME: particle mesh Ewald
RMSD: root-mean-square deviation
RMSF: root-mean-square fluctuation
RoG: radius of gyration
RRIDs: Research Resource Identifiers
SAbDab: Structural Antibody database
SVM: support vector machine
VLP: virus-like particle.
