# Supplementary material for: Computational Design of a Novel VLP-Based Vaccine for Hepatitis B Virus
Source: Front Immunol. 2020 Aug 27;11:2074. doi: 10.3389/fimmu.2020.02074 (PMC7521014; doi:10.3389/fimmu.2020.02074)
Supplement: Supplementary file 1 [file Data_Sheet_1.docx]

Supplementary Material


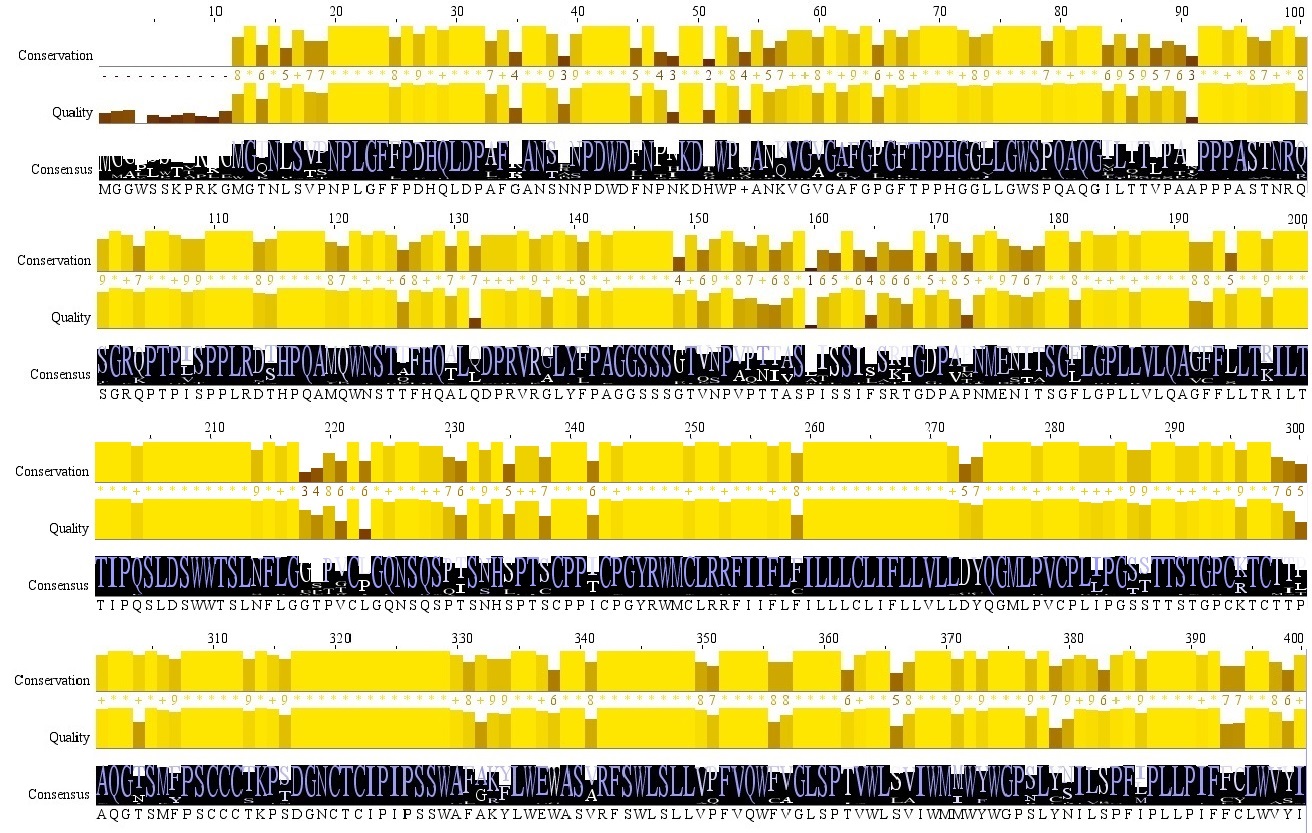


**Supplementary Figure1. Multiple sequence alignment representation of HbsAgs.** The conservation of residues is displayed as bars that higher scores indicated conserved amino acids in HBsAg sequences. The expected residues in each position is depicted in schematic view.


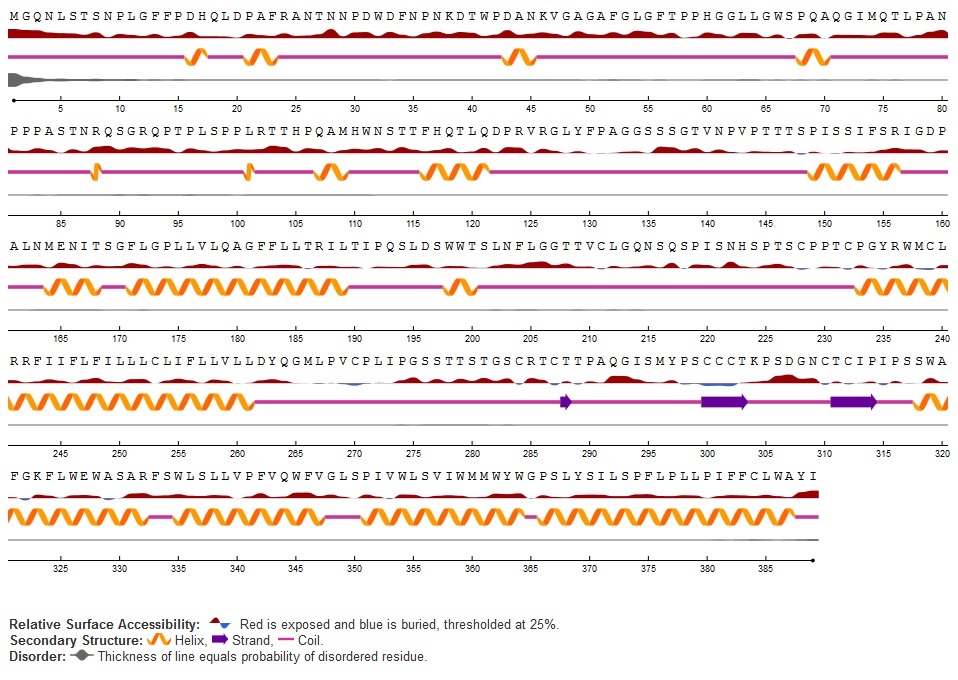


**Supplementary Figure2.** The structural feature of HBsAg protein including the surface accessibility as well as residue disorders and phi-psi dihedral angles of amino acids in the sequence, predicted by NetSurf2.0.

**
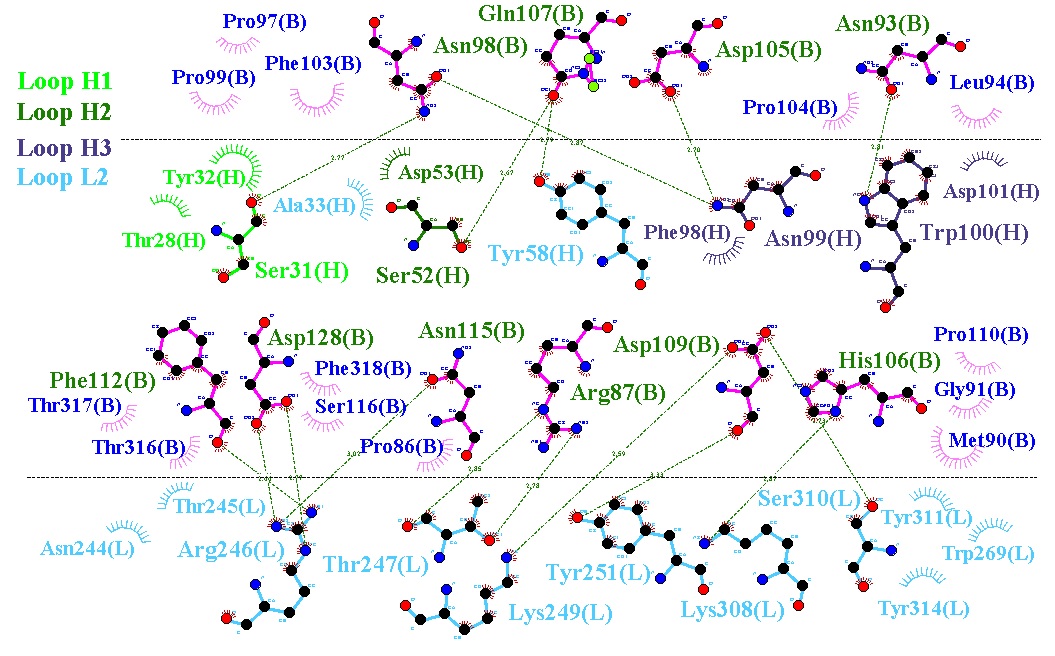
**

**Supplementary Figure3. 2D representation of 1H3P antibody complex with the Myrcludex fragment of designed vaccine.** The horizontal dashed line determines the interface of proteins in which antbody and VLP CDR residues are depicted separatly. Residues of designed vaccine are labeled in green. The CDR residues of H and L chains of antibody are coloured in light and dark green, purple and cyan, respectively. The green dashed lines between residues refer to hydrogen bonds. The non-stick residues correspond to those involved in hydrophobic contacts. The plot generated by LigPlot^+^.

**
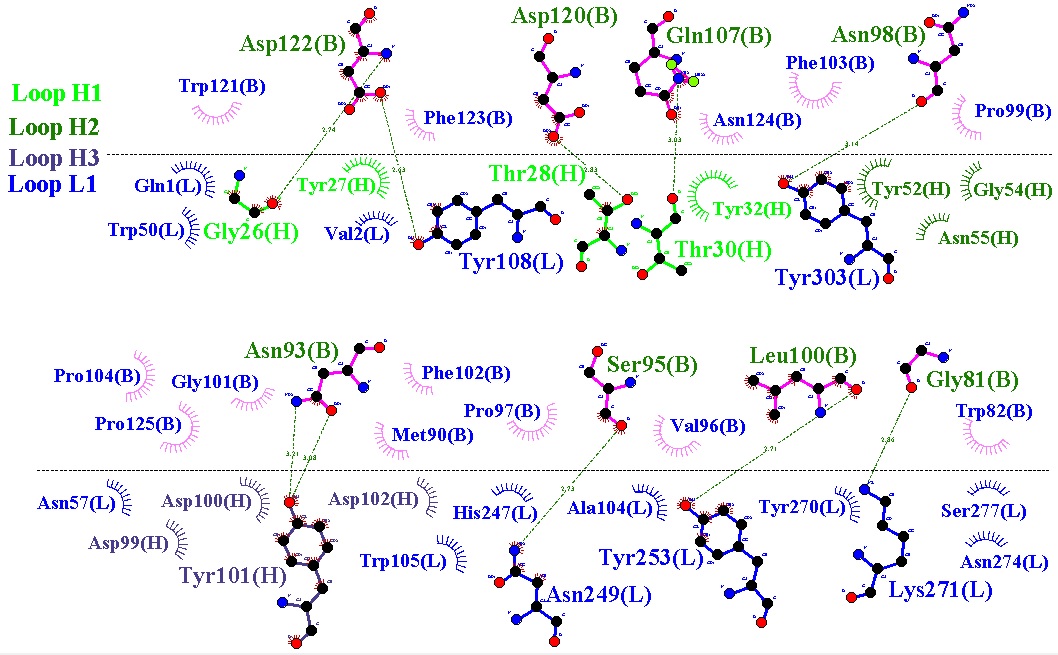
**

**Supplementary Figure4. 2D representation of 4Q0X antibody complex with the Myrcludex fragment of designed vaccine.** The horizontal dashed line determines the interface of proteins in which antbody and VLP CDR residues are depicted separatly. Residues of designed vaccine are labeled in green. The CDR residues of H and L chains of antibody are coloured in light and dark green, purple and cyan, respectively. The green dashed lines between residues refer to hydrogen bonds. The non-stick residues correspond to those involved in hydrophobic contacts. The plot generated by LigPlot+.

**
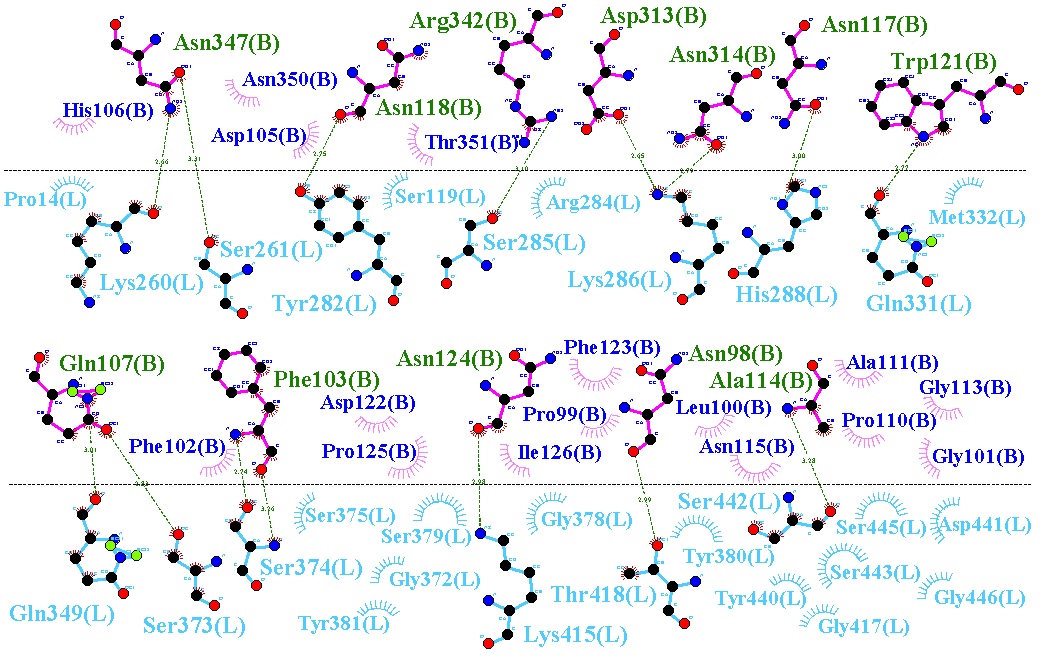
**

**Supplementary Figure5. 2D representation of 5YAX antibody complex with the Myrcludex fragment of designed vaccine.** The horizontal dashed line determines the interface of proteins in which antbody and VLP CDR residues are depicted separatly. Residues of designed vaccine and CDR region of L chain of antibody are labeled in green and cyan, respectively. The green dashed lines between residues refer to hydrogen bonds. The non-stick residues correspond to those involved in hydrophobic contacts. The plot generated by LigPlot^+^.
