# Supplementary material for: Computational Design of a Novel VLP-Based Vaccine for Hepatitis B Virus
Source: Front Immunol. 2020 Aug 27;11:2074. doi: 10.3389/fimmu.2020.02074 (PMC7521014; doi:10.3389/fimmu.2020.02074)
Supplement: Supplementary file 2 [file Data_Sheet_2.docx]

Table1. The secondary and tertiary structure validations

|  | ProSA | RAMPAGE | | | Verify 3D score % | PHD | | |
| --- | --- | --- | --- | --- | --- | --- | --- | --- |
|  | z-score | Favoured % | Allowed % | Outlier % |  | Alpha helix | Extended strand | Random coil |
| HBcAg | -2.23 | 89.2 | 8.6 | 2.2 | 82.23 | 51.08 | 11.35 | 37.57 |
| HBsAg | -4.76 | 80.7 | 15.7 | 3.6 | 80.05 | 39.46 | 10.9 | 49.64 |
| VLP-based vaccine | -2.26 | 85.6 | 11.2 | 3.2 | 80.17 | 45.63 | 12.78 | 41.59 |

Table2. Predicted MHC class I and II epitopes of the designed VLP-based vaccine.

| **HLA sets in HBV** | **MHC** | **Allele** | **Position** | **Sequence** | **Percentile rank*** |
| --- | --- | --- | --- | --- | --- |
| **Common** | MHC-I | HLA-A*02:01 | 18-27, 253-262 | FLPSDFFPSV | 0.11 |
|  | MHC-II | HLA-DRB1*01:01 | 163-177, 382-396 | ETVLEYLVSFGVWIR | 4.4 |
| **Non-responder** | MHC-I | HLA-B*08:01 | 214-222, 222-230,  431-444, 441-449 | SPRRRRSQS | 0.3 |
|  |  |  | 207-215, 426-434 | SPRRRTPSP | 0.2 |
|  |  |  | 150-158, 369-377 | LLWFHISCL | 0.5 |
|  |  |  | 142-150, 361-369 | NMGLKIRQL | 1 |
|  | MHC-II | HLA-DRB1*03:01 | 20-34, 255-269 | PSDFFPSVRDLLDTA | 7.1 |
|  |  | HLA-DRB1*07:01 | 162-176, 381-395 | RETVLEYLVSFGVWI | 2.8 |
|  |  |  | 144-158, 363-377 | GLKIRQLLWFHISCL | 7 |
|  |  | HLA-DQB1*02:01 | 11-25, 246-260 | ATVELLSFLPSDFFP | 0.77 |
|  |  |  | 5-19, 240-254 | PYKEFGATVELLSFL | 0.92 |
|  |  |  | 13-27, 248-262 | VELLSFLPSDFFPSV | 0.94 |
|  |  |  | 10-24, 245-259 | GATVELLSFLPSDFF | 0.97 |
| **Positive responsive** | MHC-II | HLA-DRB1*13:01 | 163-177, 382-396 | ETVLEYLVSFGVWIR | 0.53 |
|  |  |  | 169-183, 388-402 | LVSFGVWIRTPPAYR | 0.53 |
|  |  | HLA-DRB1*15:01 | 163-177, 382-396 | ETVLEYLVSFGVWIR | 0.84 |
|  |  | HLA-DRB1*04:01 | 132-146, 351-365 | RDLVVNYVNTNMGLK | 2.5 |

*Percentile rank ≤ 1: High affinity peptide sequences with the antigenic property

*Percentile rank ≤ 10: Intermediate affinity peptide sequences with the antigenic property

Table3. Bioinformatics tools in the study

| **Tools** | **Description** | **RRID** |
| --- | --- | --- |
| Clustal omega | Multiple Sequence Alignment (MSA) | SCR_001591 |
| MEGA7 | Phylogenetic tree | SCR_000667 |
| PHD web server | Secondary structure prediction | SCR_018778 |
| NetSurfP web server | Secondary structure prediction | SCR_018781 |
| HHPred | Structural-template alignment | SCR_010276 |
| QUARK | De novo protein structure prediction | SCR_018777 |
| Modeller | Comparative homology modeling | SCR_008395 |
| PyMol | Molecular visualization software | SCR_000305 |
| Immune Epitope Database (IEDB) | T-cell, B-cell epitope prediction | SCR_006604 |
| AlgPred web server | Allergenicity prediction | SCR_018780 |
| AllerTOP web server | Allergenicity prediction | SCR_018496 |
| VaxiJen | Antigenicity prediction | SCR_018514) |
| ANTIGENpro | Antigenicity prediction | SCR_018779 |
| C-ImmSim server | Immune simulation | SCR_018775 |
| GROMACS | Molecular Dynamics Simulations | SCR_014565 |
| ClusPro web server | Molecular docking | SCR_018248 |
| Ligplot+ software | Generation of 2D protein-protein interaction diagrams from 3D coordinates | SCR_018249 |

**Figure1. The evolutionary relation was derived using the Neighbor-Joining method and Multiple sequence alignment of HBsAg among HBV genotype D.** A) The bootstrap consensus tree concluded from 1000 replicates was taken to represent the evolutionary relation of the analyzed sequence of large envelope protein of various genotypes of HBV. The percentage of replicate trees in which the associated proteins clustered together was visible above the branches. The evolutionary distances were computed using the Poisson correction method. The analysis involved 47 amino acid sequences. Evolutionary analyses were conducted in MEGA7. B) The sequences have been colored by conservation based on Blosum62 matrix in Jalview. The most dominant genotype of HBsAg in Iran, genotype D1, is depicted by red dashlines.

**Figure2. HBsAg and HBcAg representations, the Z-score ProSA plots of structures and their cartoonic representation.** A) Schematic view of S gene in HBV. B) 3D structure of an HBsAg (residues 1–389); Myrcludex and a-determinant regions are shown in red and blue, respectively. C) 3D structure of HBcAg dimer (the MIR in monomers is shown in magenta and yellow). D-F) Secondary structure prediction of HBsAg, HBcAg dimer and designed VLP-based vaccine; Alpha helix, extended strand and random coils are indicated in blue, red and pink lines, respectively. G-I) ProSA plots of HBsAg, HBcAg and VLP-based vaccine. Black spot represents the evaluation of the input structure within the range of experimentally determined structure for proteins of similar size.

**Figure3. Structural view of designed VLP-based vaccine**. A) Overview of final stable VLP-based vaccine (insertion of 1-50 Myrcludex region of HBsAg in the first monomer of HBcAg (red) and 118-150 a-determinant of HBsAg in the second monomer (blue)). B) Structural alignment of VLP-based vaccine and HBcAg dimer in green and cyan, respectively. C-H) Close-up view of various a-determinant insertions in MIR region of second monomer; (118-150, 123-150, 121-147, 124-147, 121-148, and 123-148, respectively). I-N) Close-up view of a range of Myrcludex insertions in MIR region of first monomer; (1-50, 2-48, 1-21, 9-48, 18-48 and 20-48, respectively).

**Figure4. Predicted B‑cell epitopes of the designed VLP-based vaccine.** A) Linear B-cell epitopes based on various physicochemical properties of residues. The red boxes are related to the first and second fragments of HBsAg inserted in HBcAg. B) Predicted discontinuous B-cell epitopes for each monomer of designed vaccine.

**Figure5.** **C-ImmSim representation of immune stimulation of the designed VLP-based vaccine.** A) Phenotypic evaluation of T helper cells against a vaccine injection. B) Generation of Immunoglobulins in response to vaccine injections (0-1-6 months), specific subclasses are demonstrated as colored peaks.

**Figure6. MD analysis of HBcAg and the designed VLP-based vaccine during 100ns simulations.** A) The RMSD plot of proteins indicating acceptable stability of structures. B) The RoG plot of proteins representing stable folding of structures. C and D) The RMSF plots of HBcAg and designed vaccine, respectively. The red boxes indicated the region of inserted fragments into HBcAg.

**Figure7. Binding model of the designed VLP-based vaccine and antibodies. A**) 1H3P– designed vaccine complex; **B**) 4Q0X-designed vaccine complex and **C)** 5YAX-designed vaccine complex. The heavy and light chains of antibodies are colored in green and slate blue, respectively. The CRDs of H and L chains are displayed in magenta and yellow, respectively. The designed vaccine, Myrcludex, and ‘a’-determinant are reflected in cyan, red, and dark blue, respectively.
